# Potential Human Pathogenic Bacteria in a Mixed Urban Watershed as Revealed by Pyrosequencing

**DOI:** 10.1371/journal.pone.0079490

**Published:** 2013-11-20

**Authors:** A. Mark Ibekwe, Menu Leddy, Shelton E. Murinda

**Affiliations:** 1 United States Department of Agriculture-Agricultural Research Service, United States Salinity Laboratory, Riverside, California, United States of America; 2 Orange County Water District, Fountain Valley, California, United States of America; 3 Department of Animal and Veterinary Sciences, California State Polytechnic University, Pomona, California, United States of America; Cairo University, Egypt

## Abstract

Current microbial source tracking (MST) methods for water depend on testing for fecal indicator bacterial counts or specific marker gene sequences to identify fecal contamination where potential human pathogenic bacteria could be present. In this study, we applied 454 high-throughput pyrosequencing to identify bacterial pathogen DNA sequences, including those not traditionally monitored by MST and correlated their abundances to specific sources of contamination such as urban runoff and agricultural runoff from concentrated animal feeding operations (CAFOs), recreation park area, waste-water treatment plants, and natural sites with little or no human activities. Samples for pyrosequencing were surface water, and sediment collected from 19 sites. A total of 12,959 16S rRNA gene sequences with average length of ≤400 bp were obtained, and were assigned to corresponding taxonomic ranks using ribosomal database project (RDP), Classifier and Greengenes databases. The percent of total potential pathogens were highest in urban runoff water (7.94%), agricultural runoff sediment (6.52%), and Prado Park sediment (6.00%), respectively. Although the numbers of DNA sequence tags from pyrosequencing were very high for the natural site, corresponding percent potential pathogens were very low (3.78–4.08%). Most of the potential pathogenic bacterial sequences identified were from three major phyla, namely, *Proteobacteria*, *Bacteroidetes*, and *Firmicutes*. The use of deep sequencing may provide improved and faster methods for the identification of pathogen sources in most watersheds so that better risk assessment methods may be developed to enhance public health.

## Introduction

Traditionally, fecal indicator bacteria are used as indicators of pathogen levels of water bodies in many localities [1], instead of direct identification of individual pathogens [2]. A large number of bacteria, viruses, fungi, protists, and animalia have been identified as pathogenic for humans [3], [4] and a majority (n = 1415) are water-borne [4]. Pathogens in river water can be a problem if sewage is incompletely treated or untreated. This has been reported for many large cities in developing countries where rivers as reported for the Tietê and Pinheiros River, Brazil [5], [6] and the Ganges River in India [7], [8], are known to carry high loads of fecal bacteria. However, in developed countries, such as, the United States, Canada, and Western Europe, where sophisticated and well managed waste-water treatment facilities are available for the treatment of domestic waste; the presence of pathogenic bacteria may not be as severe as in developing countries. However, in a large, mixed, and complex watershed, there may be significant concentrations of pathogens originating from different sources feeding into the watershed.

In the Santa Ana River Watershed (southern California) there are significant amounts of water contaminants from different sources. The major sources of non-point contaminants into the river are municipal wastewater, agricultural waste discharges from dairy runoff, urban runoffs, and a combination of these sources. Currently, the Santa Ana River is impacted by one of the highest concentrations of dairy cattle in the United States. The watershed is undergoing drastic changes. In general, the varying land uses in the watershed include agriculture, open space, and rapidly growing urban areas [9]–[11]. In 1995, approximately 340 animal-confinement facilities having over 386,000 animals, mostly dairy cows, operated within the area that is mostly drained by Chino, Cypress, and Cucamonga Creeks. Pollutants in the watershed mainly consist of pathogens and nutrients due to the densely populated areas, agricultural activities, and urban and storm-water runoff in the region. Different federal, state, and private agencies have monitored fecal bacterial composition in the surface water [9]–[11], but little has been done to determine the main sources of pathogenic bacteria within the water bodies due to the complexity of the watershed. Also, the Santa Ana River is a major source of domestic water supply for over 2 million people that live in Orange County, California. The river is critical for replenishment of Orange County’s Groundwater Basin since over 2 million residents in Orange County depend on groundwater for 75% of their water needs [9]. Any factor in the watershed which degrades the river affects the quality of water for domestic water supply.

For water quality assessment, *E. coli* or enterococci are the main thermotolerant enteric bacteria commonly used to estimate the load of pathogenic bacteria in water and for microbial source tracking. Concerns have been raised about the suitability of *E. coli* or other coliform bacteria in describing the pathogenic potential of a water body [12]. For instance, the prevalence and diversity of *Salmonella* spp. (non-coliform bacteria) and their correlation with fecal pollution indicators and total heterotrophic bacteria counts were investigated in northern Greek rivers. The numbers of *Salmonella* isolates were significantly higher during summer (warm) months than winter (cold months), and the overall counts for all other microorganisms were also higher during warm months [13]. A recent Canadian study revealed a poor correlation between the numbers of coliforms and *Campylobacter* species and suggested genus-specific monitoring techniques as alternative [14]. Data on the occurrence/densities of pathogens and the impacting factors in natural waterways not only provide direct evidence of potential human health risks but also enhance predictions of the fate and transport of pathogens in surface water systems and help identify practices that reduce exposure risks [15]–[17].

In this study, the diversity and the relative abundance of pathogenic bacteria were analyzed at the genus level based on 454 pyrosequencing of bacterial 16S rRNA gene sequences. This technique has been used successfully to reveal bacterial pathogens in biosolids [18], watershed [19], and sewage-treatment plants [20]. A total of 12,959 sequences were obtained from 40 water and sediment samples, and were assigned to taxonomic ranks based on RDP Classifier and Greengenes. The overall objective of this study was to identify pathogens, including those not traditionally monitored in water and correlate their abundances to specific sources of contamination.

## Materials and Methods

### Ethics Statement

Throughout this study, normal operational procedures of the forest service and state park on the creeks and channel were followed. Permits to enter the parks and channels were obtained from the regional parks.

### Study Area and Sample Collection

This study was conducted in the middle Santa Ana River (MSAR) watershed area that covers ∼1,264 km^2^ and lies largely in the southwestern corner of San Bernardino County and northwestern corner of Riverside County and included a small part of Los Angeles County (i.e., Pomona/Claremont area) [21]. The current population of the watershed, based upon the 2000 census data, is ∼1.4 million people [10]. Land use in the MSAR watershed varies between urban and agriculture. Although originally developed as an agricultural area, the watershed is rapidly urbanizing. Open space areas include the National Forest and State Park lands. The principal remaining agricultural area in the watershed was formerly referred to as the Chino Dairy Preserve. This area is located in the south-central part of the Chino Basin sub watershed and contains approximately 200,000 dairy cows in a 77 km^2^ area (although this number is quickly declining as the rate of urban development increases) [10].

The mean annual rainfall for MSAR watershed is ≤800 mm per annum, and predominantly falls between December and April resulting in a base stream flow that is highly variable between seasons [10]. The mean annual stream flow from United states Geological Service (USGS) gauged data from Chino Creek representing urban runoff (S 3-Chino Creek @ Schaefer Ave) was 133.6 m^3^ s^−1^ and at Cypress channel representing agricultural runoff (S6– Cypress channel @ Schaefer Ave) was 96.8 m^3^ s^−1^. Sampling sites used for this study are shown in Table 1. Locations were selected for sediment and surface water sample analyses based on historical data obtained for the total maximum daily loads (TMDL) for bacterial indicators for the MSAR watershed [10]. All sampling locations, with site names, descriptions, and geographic positioning system (GPS) coordinates are listed in Table 1. Water samples at three waste-water treatment plants (WWTPs) were retrieved from the sampling ports located at the treatment plant site for sample collection (Table 1). The plants discharged tertiary-level-treated water downstream resulting in continuous but variable stream flow throughout the year along Chino Creek. Cypress Channel is more affected by dairy or agricultural runoff, and Chino Creek affected more by WWTPs and urban runoff. The Ice House Canyon (S1; Table 1), which is an open space or natural site, was used mainly as the control site because runoff from this site was mainly from melting snow. Ice House Canyon Creek is located in the San Gabriel Mountains and is a tributary to San Antonio Creek approximately 2.1 km upstream of Mt. Baldy Village. Historical data for Ice House Canyon for fecal coliforms has averaged 9 CFU 100 ml^−1^ over a five-year period, 2000 to 2005 [10]. Site M1 has the same water quality characteristics as S1, and it is at a lower elevation.

**Table 1:** Sampling locations for middle Santa Ana River pathogen source evaluation study*.

Site #	Site locations	Land use	GPS*
			Location
**S1**	Ice House Canyon	Open Space	N34°15.057 min.;W117°37.977 min; 1447 m
M1	Cucamonga Creek. @ OCWD** Ponds	Open Space	San Bernardino County Flood Control District
**S2**	Chino Creek @ Central Ave.	Urban runoff	N33°58.420 min.; W117°41.302 min;174 m***
**S3**	Chino Creek @ Schaefer Ave.	Urban runoff	N34°00.246 min.; W117°43.628 min; 207 m
**S4**	San Antonio Wash @ County Drive	Urban runoff+Commercial wash out	N30°01.543 min.; W117°43.652 min;222 m
**S5**	Chino Creek. @ Riverside Drive	Urban runoff	N34°01.144 min.; W117°44.204 min; 207 m
**S6**	Cypress Channel @ Schaefer Ave.	Agricultural Runoff	N34°00.262 min.; W117°39.766 min 208 m
**S7**	Cypress Channel @ Kimball Ave.	Agricultural Runoff	N33°58.113 min.; W117°39.624 min 177 m
**S8**	Cypress Channel @ Golf Course	Agricultural Runoff	N33°57.057 min.; W117°39.555 min;160 m
**S9**	Big League Dreams storm drain	Urban runoff	N33°57.364 min.; W117°40.788 min;163 m
S11ww	Cucamonga Creek @ RegionalWater Recycling Plant #1	Effluent from WWTP****	N34°; 01.853 min; W117°35.946 min; 246 m
S11ur	Cucamonga Creek @ RegionalWater Recycling Plant #1	Urban runoff+ wastewater	N34°; 01.853 min; W117°35.946 min; 246 m
**S12**	Chino Creek @ Pine Ave.	Urban runoff+ wastewater	N33°56.941 min.;W117°39.986 min; 155 m \
**S13**	Inland Empire Utilities Agency RegionalWater Recycling Plant #5	Effluent from WWTP	N33°57.840 min.; W117°40.826 min;180 m
**S14**	IEUA Carbon Canyon WasteReclamation Facility	Effluent from WWTP	N33°58.799 min.; W117°41.655 min;184 m elevation;
ST2	Santa Ana River @ Prado Dam	Urban Runoff	N33°; 54.737 min; W117°38.711 min 141 m.
C3	Prado Park outlet	Urban Runoff+waste discharge	N33°; 56.402 min; W117°38.763 min; 166 m
ST5	Santa Ana River @ River road	Urban Runoff	N33°; 55.405 min; W117°35.894 min; 155 m.
M5	OCWD (Prado)Wetlands Effluent	Wetland treated (bacteria loaded) OCWD	N33°; 54.737 min; W117°38.711 min; 141 m.

* Modified from Ibekwe et al. [21].

Sampling from site S10 was discontinued after one sampling due to construction activities on the site.

GPS; geographic positioning system.

OCWD; Orange County Water District.

WWTP; waste water treatment plant.

Water samples were collected using sterile Nalgene sampling bottles [22]. All samples were collected in duplicate. For sites that were deep enough to obtain samples, grab samples were collected at ∼10–15 cm below the surface of the water. Sites with a shallow flow were sampled using a sterile stainless-steel sampling device. Sediment samples from the 0- to the 10-cm depth were taken from the Creek or river banks using ethanol-disinfected core tubes and stored in Whirl-Pak bags at 4°C until processed; usually within 24 h. Field parameters consisting of electrical conductivity, pH, temperature, turbidity, and dissolved oxygen were taken at each sample location. Sample turbidity was determined using a Hach model 2100P Portable Turbidimeter (Loveland, CO) according to the manufacturer’s instructions and was calibrated daily.

### DNA Extraction and Purification from Sediment and Water Samples

Total bacterial DNA was extracted from 500 mg of sediment samples and from 250 mg pellet from a concentrated effluent sample prepared from filtered water samples after centrifugation at 3,000×g for 10 min. DNA was extracted using Power Soil and Water DNA kits (MO BIO, Inc., Solana Beach, CA), according to the manufacturer’s protocol with slight modifications. Extracted DNA (2 µL) was quantified using a Nanodrop ND-1000 spectrophotometer (Nanodrop Technologies, Wilmington DE), and run on a 1.0% agarose gel before pyrosequencing.

### Pyrosequencing

DNA samples from sediment and water were submitted to Core for Applied Genomics and Ecology (University of Nebraska Lincoln, NB) for PCR optimization and pyrosequencing analysis. The V1–V2 region of the 16S rRNA gene was amplified using bar-coded fusion primers with Roche-454 A or B titanium sequencing adapters, followed by a unique 8-base barcode sequence (B) and finally the 5′ ends of primer A-8FM (5′CCATCTCATCCCTGCGTGTCTCCGACTCAGBBBBBBBBAGAGTTTGATCMTGGCTCAG) and of primer B-357R (5′-CCTATCCCCTGTGTGCCTT GGCAGTCTCA GBBBBBBBB CTGCTGCCTYCCGTA-3′). All PCR reactions were quality-controlled for amplicon saturation by gel electrophoresis; band intensity was quantified against standards using GeneTools software (Syngene, Frederick, MD). The resulting DNA amplicon products were quantified using PicoGreen (Invitrogen, Grand Island, NY) and a Qubit fluorometer (Invitrogen, Grand Island, NY) before sequencing using Roche-454 GS FLX titanium chemistry [23], [24].

### Analysis of Pyrosequencing Data

Bacterial pyrosequencing population data were further analyzed by performing multiple sequence alignment techniques using the dist.seqs function in MOTHUR, version 1.9.1 [25]. MOTHUR was also used to assign sequences to operational taxonomic units (OTUs, 97% similarity, using the H-cluster function). Sequences were denoised using the ‘pre.cluster’ command in MOTHUR platform to remove sequences that are likely due to pyrosequencing errors [26], [27]. PCR chimeras were filtered out using Chimera Slayer [28]. Following chimera detection, the RDP Classifier function was used to assign identities to the bacterial pyrotag sequence data [29]. In addition, any sequences shorter than 400 bp in length and/or containing ambiguous base pair reads were removed from the data set.

MOTHUR was used to align the re-sampled data set and create an all-sample distance matrix, as well as assign sequences to OTUs. Overlap was calculated using the Yue-Clayton similarity estimator (θ_YC_), a metric that is scored on a scale of 0 to 1, representing absolute dissimilarity to 100 similarities [25], [30]. The metagenomic data sets of this study were deposited in Sequence read Archive under the project name SRP028870: Total bacteria from river sediment and runoff water Targeted Locus (Loci) with accession numbers SRX335804 to SRX335812 (http://www.ncbi.nlm.nih.gov/Traces/sra/sra.cgi?study=SRP028870).

## Results

### Taxonomic Assignment of the Sequences

Between 126–5,109 sequence tags (length >400 bp) were generated for each sample, resulting in 12,959 sequences and 6,462 OTUs in total from all nine sampling sites. To find the potentially pathogenic bacterial sequences from such a large amount of sequences, a reference human pathogenic bacteria list, including the species and genus names, disease caused, and the risk group (RG) was compiled using the number of sequences within 0.03 Jukes-Cantor distance of known pathogens [31]–[33] and NIH Appendix B: Classification of human etiologic agents on the basis of hazard, 2011. Although it might not be a complete list of all the human pathogenic bacteria, it covers a broad range with RG agents per NIH guidelines for human etiologic agents. The sequences obtained in this study were first assigned to proper taxonomic ranks at the genus level using RDP Classifier in MOTHUR version 1.9.1 [25], and to the species level using Greengenes. In order to check the correctness of the assignment results of the two methods, sequences from the original FASTA files were extracted, and the individual sequences RDP Classifier were searched using online BLAST (≥99%) search (http://blast.ncbi.nlm.nih.gov/blast) which is considered one of the most reliable sequence searching tools used in taxonomic studies.

### Bacterial Community Composition and Diversity

**Figure 1:**
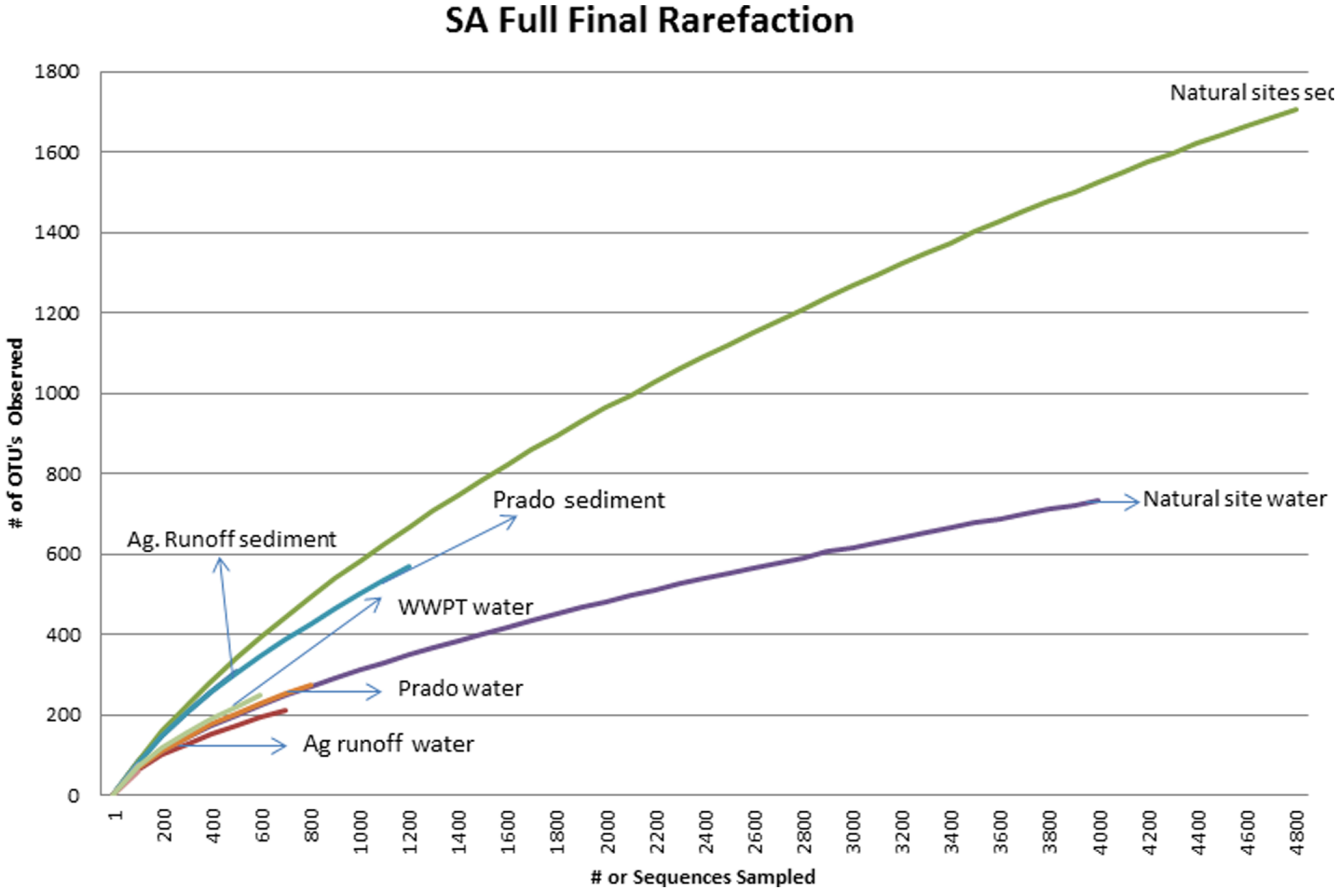
Rarefaction curves of seven sources at cutoff of 3%. Two sources (urban runoff water and sediment) are not included because of low sequence tags obtained.

**Figure 2:**
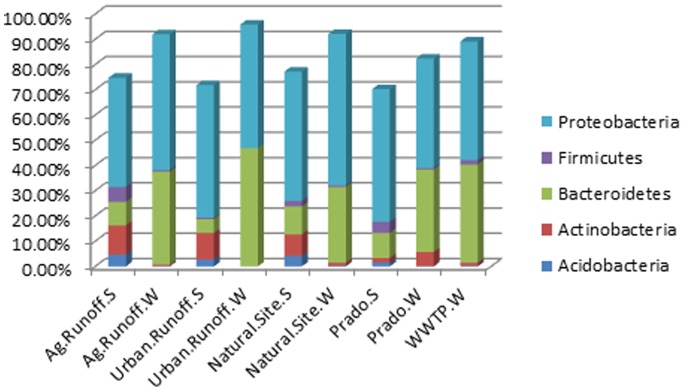
16S rRNA sequence similarity to known pathogens within each genus. The five most abundant genus are shown with their distributions within each source.

The 454 pyrosequence libraries ranged from 126 sequences from sediment samples from urban runoff to 5,109 sequences at the natural site sediment, and contained between 90 OTUs and 1,700 OTUs, respectively, as shown in the rarefaction curve (Fig. 1). Members of at least 26 bacterial phyla were detected with the 454 pyrosequencing technique. Most of the potentially pathogenic bacterial 16S rRNA encoding DNA sequences were identified from the five major phyla shown in Figure 2. *Proteobacteria* (40.73%) and *Bacteroidetes* (10.50%) were encountered most frequently. Sediments collected from sites affected by agricultural activities and the natural site contained the most diverse sequences with sequences representing the five phyla.

### Population of Potential Pathogenic Bacteria

Using RDP Classifier to identify potential pathogenic bacteria at the genus level, Table 2 shows the number of sequences identified as potentially pathogenic bacteria by 454 pyrosequencing in a mixed watershed. The relative abundance of the 36 genuses identified at the nine sites showed *Aeromonas, Clostridium, Bacillus, Pseudomonas,* and *Treponema,* as indicated in Table 2, occurred at all nine sites. However, some of these may be at the RG 1 level or opportunistic pathogens. The most dominant genus in the agricultural sediment was *Bacillus,* although this did not include any of the highly recognized pathogenic *Bacillus*.spp. However, *Aeromonas, Clostridium* and *Treponema* were the most abundant in the sediment and surface water in the natural sites and Prado Park.

**Table 2:** Number of sequence tags assigned to potential pathogenic genus from Santa Ana River watershed as determined by 454 pyrosequencing using RDP Classifier databases.

Genus	Agricultural	Agricultural	Urban	Urban	Natural	Natural	Prado	Prado	*WWT
(n = 36)_	Runoff –Sediment	Runoff –Water	Runoff –Sediment	Runoff-Water	Site –Sediment	Site –Water	Dam -Sediment	Dam -Water	P -Water
***Acholeplasma***	0	0	0	0	1	1	0	0	1
***Acinetobacter***	0	0	1	0	1	4	2	1	0
***Aeromonas***	0	4	1	3	20	32	0	1	0
***Alishewanella***	0	2	0	1	0	14	0	1	0
***Arcobacter***	3	2	0	1	3	7	2	0	1
***Bacillus***	21	0	0	0	11	1	0	2	2
***Bartonella***	0	0	0	0	1	3	1	2	1
***Borrelia***	0	0	0	0	0	0	1	0	0
***Brucella***	0	0	0	0	1	0	0	0	0
***Burkholderia***	1	0	0	0	6	3	2	0	0
***Campylobacter***	0	0	0	0	0	1	0	0	1
***Candidatus***	0	0	0	0	1	0	4	0	0
***Clostridium***	3	0	0	0	23	8	11	2	10
***Corynebacterium***	0	0	0	0	1	1	0	0	0
***Coxiella***	0	0	0	0	1	1	1	0	0
***Erysipelothrix***	0	2	1	0	13	3	19	2	1
***Escherichia***	0	1	0	0	3	0	1	0	0
***Geodermatophilus***	2	0	0	0	3	0	0	0	0
***Helicobacter***	0	0	0	0	1	1	6	0	1
***Legionella***	0	0	0	0	6	4	0	0	1
***Leptospira***	0	2	0	0	0	6	0	1	0
***Moraxella***	0	0	0	0	0	1	0	0	0
***Mycobacterium***	2	0	0	0	9	0	0	0	0
***Mycoplasma***	0	0	0	1	3	0	1	0	0
***Neochlamydia***	0	0	0	0	1	0	0	0	0
***Nocardia***	0	0	0	0	2	0	1	0	0
***Nocardioides***	0	0	1	0	23	1	1	1	0
***Pseudoalteromonas***	0	0	0	0	0	2	0	0	0
***Pseudomonas***	6	11	3	3	38	30	5	2	7
***Rickettsia***	0	5	0	1	4	4	0	0	2
***Rickettsiella***	0	0	1	0	7	1	0	1	0
***Serratia***	0	0	0	0	0	0	1	0	1
***Shewanella***	0	0	0	0	3	1	1	0	0
***Staphylococcus***	0	0	0	0	0	1	0	1	0
***Streptomyces***	1	0	0	0	10	2	1	0	0
***Treponema***	4	0	0	0	10	1	10	1	1
**Total Number** **of Samples**	660	744	178	126	5109	3460	1183	806	693
**Total Potential** **Pathogens**	43	29	8	10	206	134	71	18	30
**Potential Pathogen** **Percentages**	6.52%	3.90%	4.49%	7.94%	4.03%	3.87%	6.00%	2.23%	4.33%

* WWTP; waste water treatment plant.

We compared the sequences obtained using pyrosequencing with those of known pathogenic bacteria at the species level, and this information provided a more accurate estimation of the pathogenic populations in the samples. For each known pathogenic bacterial species, a representative 16S rRNA gene sequence was retrieved from Greengenes database (http://greengenes.lbl.gov) to create a reference database. We generated two databases; one with the RDP Classifier to the genus level and the other with the Greengenes to the species levels as recommended [34]. A total of 461 species were obtained and subjected to BLAST search to confirm their true species identity as pathogenic bacteria. Most of our FASTA sequences from 454 pyrosequencing were in agreement to the genus level with RDP Classifier. However, the Greengene identifications of sequences to the species level were not always in a 100 percent agreement with the blast search, hence we used all the data from BLAST searches as a baseline/standard.

All the sequences that were assigned to the species level as potential pathogenic bacteria from blast search are presented in Table 3. Of the 36 pathogen genus considered (Table 2), 56 species included sequences that were counted as potential pathogens with RG 2 ratings according to NIH guidelines for human etiologic agents (Table 3) [31]–[33] are discussed below.

**Table 3:** List of potential human pathogenic bacterial sequences identified from different sources within the Santa Ana watershed using 454 pyrosequencing obtained from RDP Classifier data.

Genus	Species	Source*	Riskgroup	Diseases#	PercentSimilarities	Accession #**
***Aeromonas***	*hydrophila*	N W CCWCNW	2	Gastroentreritis	98–99	NR_043638
	*veronii*	NW	2	Septicemia	99	NR_044845
***Acinetobacter***	*haemolyticus*	PW	2	Bloody diarrhea	98	NR_026207
	*junii*	NW	2	Septicemia	99	NR_026208
	*johnsonii*	CNS	2	Nosocomial	98	
***Legionella***	*pneumophila*	NW	2	Legionnaires’ Disease,Pontiac fever	96	NR_041742
	*drozanskii*	WW, NS	2	Pneumonia	97	NR_036803
	*brunensis*	NS	2	Pneumonia or flu-like illness.	93	NR_026520
	*impletisoli*	NS	2	Pneumonia or flu-like illness	94	NR_041321
	*drancourtii*	NW	2	Pneumonia	93	NR_026335
***Bartonella***	*chomelii*	NS	2	Cat scratch fever by henselae	87	NR_025736
***Brucella***	*microti*	NS	3	Brucellosis	96	NR_042549
***Burkholderia***	*mimosarum*	PS	2	Nonpathogenic	98	NR_043167
***Clostridium***	*rectum*	NW, PS	2	Gastroenteritis??	94–95	NR_029271
	*cocleatum*	NW	2	Nonpathogenic	99	NR_026495
	*acidisoli*	NS	2	Nonpathogenic	98	NR_028898
	*cellobioparum*	NW	2	Nonpathogenic	98	NR_026104
	*bartlettii*	NW, WW, NS	2	Nonpathogenic	99	NR_027573
	*hiranonis*	WW	2	Nonpathogenic	99	NR_028611
	*irregulare*	WW	2		92	NR_029249
	*cellulovorans*	NS, PS	2	Nonpathogenic	97	NR_027589
	*aciditolerans*	PS, NS	2	Nonpathogenic	98	NR_043557
	*thermobutyricum*	PS	2	Nonpathogenic	97	NR_044849
	*sulfidigenes*	NS3	2	Nonpathogenic	96–99	NR_044161
	*clariflavum*	CCS	2	Nonpathogenic	90	NR_041235
	*citroniae*	NS	2	From unspecified clinicalinfections (likely nonpatho.)	96	NR_043681
	*disporicum*	NS	2	Bacteraemia	95	NR_026491
***Corynebacterium***	*appendicis*	NW	2	Appendicitis*	98	NR_028951
	*callunae*	NS	2		97	NR_037036
***Erysipelothrix***	*inopinata*	CCW, PS,PW, NS, NW	2	Nonpathogenic	92–93	NR_025594
***Escherichia***	*albertii*	NS	2	diarrheal disease	99	NR_025569
***Helicobacter***	*brantae*	PS		Nonpathogenic	100	NR_043799
***Leptospira***	*meyeri*	NW	2	Unclear role/potentially	94–95	NR_043045
	*wolbachii*	CCW	2	Nonpathogenic	99	NR_043046
	*alexanderi*	NW	2	Pathogenic to animals	94	NR_043047
***Mycobacterium***	*moriokaense*	NS	2	Pneumonia	98	NR_025526
	*brumae*	CCS	2	Bacteraema	96	NR_025233
	*aurum*	NS	2	Bacteraemia, keratitis	99	NR_029217
	*pallens*	NS	2	Not available	99	NR_043760
	*tusciae*	NS	2	Lymphnode/chronic fibrosis	99	NR_024903
	*farcinogenes*	NS	2	Prosthesis infection	99	NR_042923
***Nocardia***	*nova*	NS	2	Nocardiosis	99	NR_041858
***Rickettsia***	*montanensis*	NW	3	Nonpathogenic for humans	90	NR_025920
	*aeschlimannii*	NW	3	Tickborne rikettsiosis	98	NR_026042
	*asiatica*	WW	3	Unknown pathogenesis for humans	87	NR_041840
	*canadensis*	NS	3	Febrile disease	87	NR_029155
	*raoultii*	NS	3	Tickborne lympanopathy	93	NR_043755
***Salmonella***	*enterica*	NS	2	Gastroenteritis	98	NR_044373
***Serratia***	*liquefaciens*	ww	2	Gastroenteritis	95	NR_042062
***Staphylococcus***	*aureus*	NW	2	Gastroenteritis, skin infections	100	NR_037007
***Treponema***	*primitia*	PW, PS,CNS, NS	2	Syphilis, yaws	92	NR_041714
	*denticola*	CCW	2	Periodontal disease	92	NR_036899
	*azotonutricium*	PS, CNS	2	Nonhuman pathogenic	90	NR_025141
	*berlinense*	NS	2	Nonpathogenic	85	NR_042797
	*medium*	NS		Periodontal disease	91–92	NR_037137

N = Natural site; W = Water, S = sediment, CC = Cypress Channel, CN = Chino Creek; P = Prado wetland area: e.g. NS = Natural site sediment.

*Mycobacteria, Ligionella, Treponema,* and *Clostridia* were the most common, with different species and were the only pathogen present in most of the sites sampled. The most common *Mycobacteria* spp were the opportunistic pathogens, *M. moriokaense, M. farcinogenes, M. brumae, M. aurum, M. pallens, and M. tusciae*. All except *M. brumae* that was found in Chino Creek sediment, which is impacted by urban runoff, were found in natural site sediments. The most common potentially pathogenic *Clostridia* spp. was *C. bartlettii* which was isolated from natural site water and in WWTP. Most of our sequences had very high similarity levels with known human pathogens (Table 3). There were many other species of potentially pathogenic bacterial sequences that were recovered from our samples. Most notable were two sequences that were 96% similar to *Brucella microti* and 87–98% similar to *Rickettsia* spp. which are both RG 3 pathogens (Table 3). *The Brucella microti* sequences were recovered from natural site sediments while those of *Rickettsia spp* were recovered from both water and sediment samples from natural sites and WWTP.

## Discussion

Total potential pathogens (%) were highest in urban runoff water (7.94%), agricultural runoff sediment (6.52%) and Prado Park sediment (6.00%), respectively (Table 2). Although the numbers of sequence tags from 454 were very high for the natural site, percent potential pathogens were very low. The higher percent potential pathogen in urban runoff water is a very serious concern. Most of our urban runoff and agricultural runoff samples contained opportunistic pathogens that are common in soil such as (*Clostridia*, *Mycobacteria*, and *Nocardia* spp.). One human pathogen *Staphylococcus* spp. was found mainly in the natural sites and Prado Park, and *Staphylococcus aureus* sequence in the natural site were confirmed by BLAST searches. However, two sequences of *Legionella pneumophila* were recovered from water samples collected from the natural site. It should be noted that *L. pneumophila* causes about 90% of all reported cases of legionellosis in the United States [35]. Water is the major reservoir for legionellae, and the bacteria are found in freshwater environments worldwide. In another study legionellae were detected in 40% of freshwater environments by culture and in 80% of these samples by PCR [36].

Sequences from *Mycobacterium* genus had a relatively high similarity (96–99%) to the known pathogenic species in this genus. Most of the sequences uncovered in this study were from sediment samples, which was contrary to our expectations because *Mycobacterium* spp. are known to be very common in water samples. Two other genus that were found in many samples and with different species representatives were *Aeromonas* and *Treponema*. *Aeromonas* spp. were found in water samples from the natural site, WWPTs, and Chino Creek which is impacted by urban runoff. The most common species sequences from the Creek samples was *Aeromonas hydrophila* which is known to be very toxic to many organisms because it produces Aerolysin Cytotoxic Enterotoxin (ACT), a toxin that can cause tissue damage [37]. Five *Treponema* spp sequences were found (Table 2), and this pathogen had one of the most widespread sequences which were found in 6 of 9 sources throughout the watershed. These pathogens are mainly anaerobic, fastidious, highly mobile, and are found in the oral cavity, digestive track and genital areas of human, animals, and insects [38]. Several species of this pathogen are associated with syphilis in human, human periodontal infection, and bovine digital dermatitis [38]. The diversities and abundances of different genus were quite distinct at the natural sites, indicating potential pathogenic bacteria at this site, despites the lack of inputs from contaminants. This may be dominated by sequences from organisms with many generations.

As the goal of this study was to identify pathogens, including those that are not traditionally monitored, the sequencing depth necessary to identify these pathogens was not known prior to the study. With the goal of directing future monitoring and risk assessment efforts, the sample sites selected were those that were previously used for TMDL evaluation study [10]. The pathogenic bacterial sequences identified in this study include some of the most common bacteria that are very pathogenic and could be used for microbial source tracking such as *Clostridia*, *Rickettsia*, and *Brucella* spp. It should be noted that we did not identify sequences that belong to some of the most pathogenic *Clostridia* spp. such as *C. botulinum*, *C. difficile*, *C. perfringens*, *C. tetani*, and *C. sordellii*. *Clostridium* consists of around 100 species that include common free-living bacteria as well as important pathogens [39]. Only five sequences that were 93–98% similar to *Rickettsia* spp. and two sequences that were 96–97 similar to *Brucella microti* were identified in this study. These are RG 3 agents that are associated with serious or lethal human disease for which preventive or therapeutic interventions may be available in comparison to RG 4 agents for which preventative or therapeutic interventions are not available. It should be noted that *Rickettsia* spp. are carried by many ticks, fleas, and lice, and cause diseases in humans, such as, typhus, rickettsial pox, Boutonneuse fever, African tick bite fever, Rocky Mountain spotted fever, Flinders Island spotted fever and Queensland tick typhus [40]. Most of our *Rickettsia* spp. sequences were identified from natural site sediment and water and from WWTPs effluent. However, the two *B. microti* were recovered from natural site sediment. This confirmed what has been recently reported that *B. microti* may be very common in soils [41].

Some sequences of the more common foodborne pathogens were found in relatively low numbers. These include Escherichia and Salmonella genus. Two sequences with 99% similarity belonging to *Escherichia albertii* were found in the natural site sediment and two sequences with 98% similarities for *Salmonella enterica* were found in the natural sites. *E. albertii* is a potential human foodborne pathogen because of its documented ability to cause diarrheal disease by producing attachment and effacement lesions. It can tolerate heat (56°C), acid (pH 3.0), and hydrostatic pressure (500 MPa) [42]. *Escherichia* and *Salmonella* are common animal pathogens [43]. *Helicobacter* were isolated from Prado Park water sediment while *Leptospira* spp. were isolated from samples from the natural sites, and the Cypress channel which is impacted by dairy farm runoff. Leptospirosis is among the world’s most common diseases transmitted to people from animals via urine-contaminated water that comes in contact with unhealed breaks in the skin, the eyes, or with the mucous membranes [44]. It was not surprising, therefore, that most of the *Leptospira* spp. sequences were found in water samples impacted by dairy runoff.

In this study, we employed high-throughput 454 pyrosequencing technique to quantify bacterial community structure in a large watershed impacted by many pollutant sources such as 11 WWTPs, large urban population of about 1.4 million and a large cattle operation. As far as we know, this is the first effort to use this technique to detect human bacterial pathogens in a large watershed. Although not as sensitive as qPCR at the current sequencing practice, this technique may overcome the limitation of the PCR-based detection techniques, which may introduce nonspecific amplification and highly relies on the primers selected. Another advantage for this technique is high-throughput, which can target community composition [45] and all concerned pathogens in a single detection. As shown in this study, this technique mainly contained pyrosequencing and bioinformatic analysis, which showed a comprehensive profile of detected bacterial pathogens within the watershed. It serves as a powerful and promising approach to monitor and track human bacterial pathogens. However, it is worthy to note that such molecular technique is difficult to exactly quantify pathogens in terms of cell number in surface water or sediment because of the high complexity to convert gene copy number to cell number.

## Conclusion

The main objective of this study was to identify potential sources of pathogens in the environment (i.e., mixed urban watershed) with respect to human exposure and risk. Based on deep sequencing, we were able to identify sources of potential pathogens belonging to RG 1, RG 2 and RG 3. This presents an added advantage because these pathogens could be further enriched and studied further or quantified using real-time PCR after designing primers at the genus level to focus on the quantification of pathogens with potential risk to human public health. This could lead to better understanding of pathogen loads in the environment and enabling of more effective assessment of their fate and transport in the environment.
